# Identifying meaningful change on PROMIS short forms in cancer patients: a comparison of item response theory and classic test theory frameworks

**DOI:** 10.1007/s11136-022-03255-3

**Published:** 2022-09-24

**Authors:** Minji K. Lee, John D. Peipert, David Cella, Kathleen J. Yost, David T. Eton, Paul J. Novotny, Jeff A. Sloan, Amylou C. Dueck

**Affiliations:** 1 Department of Quantitative Health Sciences, Mayo Clinic, 200 First Street SW, Rochester, MN 55905 USA; 2 Department of Medical Social Sciences, Northwestern University Feinberg School of Medicine, 625 Michigan Ave, 27th Floor, Chicago, IL 60611 USA; 3 Department of Quantitative Health Sciences, Mayo Clinic, 13400 E. Shea Blvd., Scottsdale, AZ 85259 USA

**Keywords:** Reliable change index, Item Response Theory, Classical Test Theory, Patient Reported Outcomes Measurement Information System, Meaningful change, PRO-CTCAE

## Abstract

**Background:**

This study compares classical test theory and item response theory frameworks to determine reliable change. Reliable change followed by anchoring to the change in categorically distinct responses on a criterion measure is a useful method to detect meaningful change on a target measure.

**Methods:**

Adult cancer patients were recruited from five cancer centers. Baseline and follow-up assessments at 6 weeks were administered. We investigated short forms derived from PROMIS® item banks on anxiety, depression, fatigue, pain intensity, pain interference, and sleep disturbance. We detected reliable change using reliable change index (RCI). We derived the T-scores corresponding to the RCI calculated under IRT and CTT frameworks using PROMIS® short forms. For changes that were reliable, meaningful change was identified using patient-reported change in PRO-CTCAE by at least one level. For both CTT and IRT approaches, we applied one-sided tests to detect reliable improvement or worsening using RCI. We compared the percentages of patients with reliable change and reliable/meaningful change.

**Results:**

The amount of change in *T* score corresponding to RCI_CTT_ of 1.65 ranged from 5.1 to 9.2 depending on domains. The amount of change corresponding to RCI_IRT_ of 1.65 varied across the score range, and the minimum change ranged from 3.0 to 8.2 depending on domains. Across domains, the RCI_CTT_ and RCI_IRT_ classified 80% to 98% of the patients consistently. When there was disagreement, the RCI_IRT_ tended to identify more patients as having reliably changed compared to RCI_CTT_ if scores at both timepoints were in the range of 43 to 78 in anxiety, 45 to 70 in depression, 38 to 80 in fatigue, 35 to 78 in sleep disturbance, and 48 to 74 in pain interference, due to smaller standard errors in these ranges using the IRT method. The CTT method found more changes compared to IRT for the pain intensity domain that was shorter in length. Using RCI_CTT_, 22% to 66% had reliable change in either direction depending on domains, and among these patients, 62% to 83% had meaningful change. Using RCI_IRT_, 37% to 68% had reliable change in either direction, and among these patients, 62% to 81% had meaningful change.

**Conclusion:**

Applying the two-step criteria demonstrated in this study, we determined how much change is needed to declare reliable change at different levels of baseline scores. We offer reference values for percentage of patients who meaningfully change for investigators using the PROMIS instruments in oncology.

**Supplementary Information:**

The online version contains supplementary material available at 10.1007/s11136-022-03255-3.

## Background

The determination of change in patients undergoing an intervention is an important element in clinical trials when researchers are interested in progress or improvement. Beyond clinical trial setting, providing personalized feedback to care providers based on patients’ change in symptoms is becoming more relevant as the electronic health system enables routine collection and analysis of patient-reported outcomes (PROs) on a real-time basis. Identifying patients who had reliable changes in their scores can lead to more valid interpretation and communication of the change and opportunities for clinical action. Change can be studied at the population-level or individual-level: The population-level change focuses on the population parameters (e.g., means) over time, while individual-level change focuses on change in an individual’s score over time [1]. It has been suggested that ‘responders’ to treatment need to be identified based on the significance of individual change using indices such as reliable change index (RCI) rather than group-level change[2].

Clinically meaningful change can be discussed only after we determine that an observed change is reliable [3]. It would not be logical to speak of whether a change is clinically meaningful unless we can be confident that change has occurred [4]. RCI is a method for determining the statistical significance of an observed change in a single patient, which is expressed as a ratio of the observed change for an individual to nuisance effects. The RCI indicates whether an individual’s observed change “reflects more than the fluctuations of an imprecise measuring instrument” [5]. Of note, RCI traditionally investigated within-person change using between-person statistic, which garnered some attention regarding whether it should use within-individual statistic instead. Though the denominator of the RCI contains group-level statistics, the numerator only contains information about the individual. In this way, the RCI indexes the raw change in the individual to the variability of the group as well as the reliability of the measure, offering an advantage over a raw change score only. In addition, meaningfulness of clinical data such as a laboratory value is not generated using within-individual statistics because such standards would not be accurate at the individual level. Rather, establishing clinical thresholds often relies on distributional data such as probabilities from grouped data or between-subject analyses with defined clinical groups. Similar group-level methods have been applied for PROs to set thresholds for interpreting individual-level PRO data [6], which can lead to increased use of PROs in clinical care.

Recently, there have been efforts to study RCI using IRT statistics. These studies noted that IRT-based methods consider the difference in measurement precision across the scale whereas the classical RCI uses a fixed standard error of measurement (SEM) [7; 8]. Mellenbergh [1] stated that the IRT methods may be more ‘person-oriented’ than the observed score method based on classical test theory (CTT). In parametric IRT, the concept of reliability is replaced by test information [9]. Items with larger discrimination parameters provide more information at the location indicated by item location parameter. Test information is the sum of item information, and the standard error of estimation in IRT is inversely related to the test information function, which shows that we may have different standard errors depending on how discriminating the set of items are for ranges of attributes measured. Prior literature focused on describing the differences in identification rates between CTT-based and IRT-based RCI statistics. Brower et al.[7] and Jones et al. [10] showed that most people were classified consistently between IRT-based RCI (RCI_IRT_) and CTT-based RCI (RCI_CTT_). Jabrayilov et al. [11] reported that IRT detects more people as having changed compared to CTT, provided that tests contain many items (e.g., 20 items), whereas for shorter tests (e.g., 5 items), CTT better detects change than IRT. Using Patient Reported Outcomes Measurement Information System-29 (PROMIS-29) physical and emotional distress scales, Hays, Spritzer, and Reise [8] found CTT identified more people as having changed compared to IRT.

The motivation for using IRT-based computer adaptive testing (CAT) is an accurate estimation of true scores with items tailored to patients. The accuracy and efficiency result from using the items with maximum information where patients’ true scores lie, and this way, patients can be administered a smaller number of items. Because IRT-based assessments rely on item/test information, it is natural to use standard error of estimation from IRT to assess the reliability of change. The current study investigates the effect of IRT-based standard errors and CTT-based standard errors on identification of reliable change. In addition, we identify patients who had meaningful change in several PROMIS scales using the categorical information provided by single-item measures that directly communicate patients’ categorization of their own symptoms (e.g., PRO-CTCAE: PRO version of the Common Terminology Criteria for Adverse Events).

Unlike CTT, IRT methods do not require pretest and posttest measurements to be based on the same items as long as all items are calibrated on the same scale [11]. However, to fairly compare CTT and IRT, the same items from PROMIS short forms were used at baseline and follow-up. One can fit IRT models to PRO measures that are not built with IRT but this requires additional investigation into model assumptions and model fit. In this study, we used PROMIS because it is well-received in terms of robust IRT parameters based on a large sample representative of the U.S. census, which allows us to focus on our primary objective of the study, the comparison of classifications between CTT and IRT.

Our research questions are as follows:

1. What percentages of patients had reliable improvement and worsening based on CTT and IRT using PROMIS short forms?
2. What is the magnitude of the reliable change scores based on CTT and IRT methods on PROMIS short forms?
3. Among the patients who had reliable change in PROMIS short forms, what percentage also had meaningful change based on CTT and IRT?

## Methods

### Sample

Adult cancer patients were recruited from five cancer centers: University of North Carolina, Memorial Sloan-Kettering Cancer Center, Northwestern University, MD Anderson Cancer Center, and Mayo Clinic in Rochester, Minnesota. Patients were eligible for the study if they had a diagnosis of cancer, were currently receiving anti-cancer treatment or would be initiating active anti-cancer treatment within the next seven days or underwent surgery for cancer treatment in the past 14 days. Patients treated with only hormonal therapy and patients with clinically significant cognitive impairment were excluded. The study was reviewed by the IRB of each of the participating sites, and all patients provided consent to enter the study. Baseline and 6-week follow-up assessments were administered.

### Measures

We investigated six version 1.0 short forms derived from PROMIS item banks: Anxiety 8a, Depression 8a, Fatigue 7a with two additional items from Fatigue 8a, Sleep Disturbance 8a, Pain Intensity 3a, and Pain Interference 8a. All scales had eight to nine items, but pain intensity. Pain intensity scale had three items, which let us examine the effect of test length on differences in classifications by CTT and IRT. The PROMIS measures are scored on a *T* score metric in which 50 is the mean of a general US adult reference population and 10 is the standard deviation (SD) of that reference population.

PRO-CTCAE items provide categorical information on patient symptom levels and are used in this study for evaluating meaningful change. PRO-CTCAE mirrors clinician adverse event reporting (CTCAE), was developed with patient and clinician input [12; 13] and has validity and reliability evidence in cancer samples [14; 15]. PRO-CTCAE has also been used as clinical decision support, in which the extreme response categories of “severe”/ “very severe”, “quite a bit”/ “very much”, or “frequently”/ “almost always” trigger nurse alert [16]. PRO-CTCAE items were available for all six domains and they had five response options such as none, mild, moderate, severe, and very severe. Because each CTCAE grade can inform clinical actions (e.g., In dehydration [17], grade 1 = increased oral fluids indicated, grade 2 = IV fluids indicated, grade 3 = hospitalization indicated, grade 4 = urgent intervention indicated) and based on the study that found each ordinal response choice in PRO-CTCAE served to distinguish respondents with meaningfully different symptom experiences, any 1-level change in the PRO version of CTCAE (PRO-CTCAE) was considered meaningful in this study.

Because the response options used in Anxiety 8a and Depression 8a were “never”, “rarely”, “sometimes”, “often”, and “always”, we used corresponding PRO-CTCAE frequency items (“In the past 7 days, how often did you feel anxiety?” and “In the past 7 days, how often did you have sad or unhappy feelings?”). Although Fatigue 7a + 2 had frequency-based response options, PRO-CTCAE does not have a frequency item for fatigue, so we used PRO-CTCAE fatigue severity item instead (“In the past 7 days, what was severity of your fatigue, tiredness, or lack of energy at its worst?”). PROMIS Sleep Disturbance 8a asked sleep quality where higher response options indicated greater disturbance, so we used the PRO-CTCAE severity item (“In the past 7 days, what was the severity of your insomnia including difficulty falling asleep, staying asleep, or waking up early at its worst?”). We used the PRO-CTCAE severity and interference items for the corresponding PROMIS Pain Intensity 3a, and Pain Interference 8a.

### Defining reliable change

In the literature on clinically important patient-level changes, the RCI has served as cutoff for individual-level change indicating whether the observed change was of sufficient magnitude to exceed the margin of measurement error. The RCI lets us test the null hypothesis that there was no change between measurements. We used a one-tailed test, in which an RCI value exceeding |1.65| indicates reliable improvement or deterioration has occurred. For the CTT approach, the RCI_CTT_ is calculated as

1 $$\mathrm{RCICTT}=\frac{{x}_{2}-{x}_{1}}{\sqrt{{SEM}_{2}^{2}+{SEM}_{1}^{2}}}$$

where ${x}_{2}$ is the posttest score, ${x}_{1}$ is the pretest score, and SEM_2_ is the SEM of the posttest score, and SEM_1_ is the SEM of the pretest score. For the pretest and posttest scores, we used the *T* scores rather than sum scores to reflect the actual reporting metric. SEM is calculated as the SD of either the pretest or posttest score multiplied by the square root of one minus the reliability of the PROMIS short form. In alternative formulations, the denominator can also be computed based on the SEM of the pretest score only (i.e., $\sqrt{2\times {SEM}_{1}^{2}}$) but we used the standard errors at baseline and follow-up rather than just the baseline SEM, in which the equality of pre and posttest variances is not assumed [5; 18; 19], to fairly compare with the RCI_IRT_.

For reliability, we use McDonald’s coefficient omega (hierarchical) (${\omega }_{h}$) as an estimate of the general factor saturation of a test [20] using R package ‘psych’ [21]. This conceptualization of reliability (i.e., proportion of variance in the scale scores accounted for by a general factor) is consistent with the unidimensional IRT model that we use for computing RCI_IRT_. Zinbarg and others [22] compared McDonald’s ${\omega }_{h}$ to Cronbach’s *α*, and concluded ${\omega }_{h}$ is a better estimate, because Cronbach’s *α* reflects not only general factor saturation but also group factor saturation and even variability in factor loadings. Note that in a truly unidimensional test, Cronbach’s *α* will be very close in value to ${\omega }_{h}$. We extracted three group factors in addition to the general factor when estimating ${\omega }_{h}$. For pain intensity, we extracted only a general factor, because there were only three items. Although we use ${\omega }_{h}$ to compute RCI_CTT_, we also report Cronbach’s *α* in descriptive statistics for comparison.

IRT provides a statistic, the standard error of estimation or $SE(\widehat{\uptheta })$, that varies conditionally on trait level, and is inversely related to the amount of information provided by an instrument. The magnitude of the standard error depends on the persons’ location and whether items are close to this location on a latent continuum. Thus, this standard error depends on item parameters and the number of items the person has been administered. The RCI in the context of IRT [7; 11] can be defined as

2 $$\mathrm{RCIIRT}=\frac{{x}_{2}-{x}_{1}}{\sqrt{{SE({x}_{1})}^{2}+{SE({x}_{2})}^{2}}}$$

where ${x}_{2}$ is the posttest score, ${x}_{1}$ is the pretest score on T-score metric, SE(x_1_) and SE(x_2_) are the standard errors of estimation multiplied by 10 at baseline and follow-up (to match the T-score metric), respectively. Standard errors are estimated using expected a posteriori (EAP) estimation. EAP scoring is employed to estimate scores for the vast majority of PROMIS measures due to its attractive properties in the context of computer adaptive testing, especially around test termination [23]. Because we use *T* scores that are based on IRT person estimates in both RCI_CTT_ and RCI_IRT_, the only difference between the decisions come down to the different methods of computing standard errors. Because we subtract the pretest score from the posttest score where higher scores indicate more severe symptoms, a positive RCI value indicates the change is in the direction of getting worse and a negative RCI value indicates the direction of getting better.

We used one-tailed tests of significance in the current study. We felt that worsening and improvement need to be examined separately, without assuming the percentages identified as reliably changed are equal between improvement and worsening. For both RCI_CTT_ and RCI_IRT_, an RCI value greater than |1.65| classifies a patient getting either better or worse.

### Identifying patients who had reliable change and meaningful change

The degree of agreement in classifying patients as having experienced change between CTT and IRT methods was expressed in terms of sample sizes and percentages. Among the patients who had reliable change, we further identified patients with meaningful change.

## Results

### Sample

For each of the scales, we analyzed the scores of adult patients recruited from five cancer centers who had complete responses for both baseline and follow-up data. There were originally 1,859 patients, and after selecting patients with complete responses for both baseline and follow-up assessments, the sample sizes for the 5 scales ranged from 1,089 to 1,162 (Table 1). The demographic information on the full sample at baseline (n = 1,859) has been previously described [24]. There were 1,253 patients who had PROMIS change scores available in any of the six scales. The age of the 1,253 patients ranged from 18 to 89 with the median, 58. There were 907 White (72%), 255 Black (20%), 38 Asian (3%), and 9 American Indian/Alaska Native (0.3%) patients. There were 57 Hispanic/Latino patients (5%). Cancer types included breast (313, 25%), lymphoma/myeloma (279, 22%), prostate/bladder (16, 1%), lung (92, 7%), colorectal (115, 9%), head/neck/gastroesophageal (95, 8%), and other (307, 25%). In terms of clinician-reported Eastern Cooperative Oncology Group (ECOG) performance status (PS), 602 (48%) had normal activities without symptoms (score of 0), 557 (44%) had some symptoms but did not require bed rest during waking day (score of 1), 86 (7%) required bed rest for less than 50% of waking day (score of 2), and 8 (1%) required best rest for more than 50% of waking day (score of 3). In terms of patient-reported ECOG, 304 (24%) reported 0, 592 (47%) reported 1, 279 (22%) reported 2, 44 (4%) reported 3, and 1 (0.08%) reported 4 (totally disabled; totally confined to bed or chair). The distribution of the ECOG at the follow-up was similar to that of the baseline. In terms of disease stage, 154 (12%) patients were stage I, 261 (21%) stage II, 367 (29%) stage III, and 414 (33%) stage IV.

**Table 1:** Descriptive statistics of the baseline scores, 6-week follow-up scores, and the change scores

	Baseline	Follow-up	Change (Follow-up – Baseline) + : Worsening −: Improvement
*Anxiety (n = 1,105)*			
Min, Median, Max of T score	37, 49, 78	37, 51, 83	−29, 0, 39
Mean (SD) of **T score**	48.7 (9.2)	50.0 (9.9)	1.2 (8.6)
Min, Median, Max of **SE**	1.8, 2.3, 5.5	1.8, 2.2, 5.5	
Mean (SD) of **SE**	3.2 (1.5)	3.1 (1.5)	
*Depression (n = 1,162)*			
Min, Median, Max of **T score**	38, 47, 81	38, 48, 81	−32, 0, 36
Mean (SD) of **T score**	47.1 (8.3)	48.4 (9.3)	1.2 (8.0)
Min, Median, Max of **SE**	1.5, 2.5, 5.7	1.5, 2.4, 5.7	
Mean (SD) of **SE**	3.5 (1.7)	3.4 (1.8)	
*Fatigue (n = 1,140)*			
Min, Median, Max of **T score**	29, 52, 77	29, 54, 81	−23, 2, 31
Mean (SD) of **T score**	51.2 (8.7)	53.8 (8.8)	2.6 (8.0)
Min, Median, Max of **SE**	2.0, 2.1, 5.2	2.0, 2.1, 5.2	
Mean (SD) of **SE**	2.3 (0.5)	2.2 (0.4)	
*Pain Interference (n = 1,090)*			
Min, Median, Max of **T score**	41, 51, 77	41, 53, 77	−27, 0, 37
Mean (SD) of **T score**	50.3 (9.5)	51.3 (10.3)	1.0 (9.4)
Min, Median, Max of **SE**	1.2, 1.8, 5.9	1.2, 1.6, 5.9	
Mean (SD) of **SE**	3.3 (2.2)	3.3 (2.2)	
*Pain Intensity (n = 1,144)*			
Min, Median, Max of **T score**	36, 48, 81	36, 50, 82	−36.5, 0, 46
Mean (SD) of **T score**	48.1 (10.5)	48.9 (10.9)	0.8 (10.0)
Min, Median, Max of **SE**	3.3, 3.6, 6.2	3.3, 3.6, 5.4	
Mean (SD) of **SE**	4.2 (0.9)	4.2, (0.9)	
*Sleep Disturbance (n = 1,089)*			
Min, Median, Max of **T score**	31, 49, 78	31, 51, 78	−30, 0.6, 32
Mean (SD) of **T score**	49.1 (9.2)	50.5 (9.4)	1.4 (8.6)
Min, Median, Max of **SE**	2.1, 2.6, 4.9	2.1, 2.5, 4.9	
Mean (SD) of **SE**	2.8 (0.7)	2.7 (0.6)	

### Descriptive statistics

Table 1 shows the descriptive statistics of the six measures, where higher scores indicate worse symptoms. The mean baseline scores ranged from 47 to 51 across measures. The mean follow-up scores ranged from 48 to 54. The largest change was in fatigue with the average change of 2.6 (deterioration). The mean change scores ranged from 0.8 to 2.6 across measures.

For reliability estimates, ${\omega }_{h}$ and the coefficient *α* were greater than 0.80 across scales. In addition, the ${\omega }_{h}$ and the coefficient *α* values were similar, suggesting that the scales could be mostly explained by their respective general factor (Table 2).

**Table 2:** Reliability estimates, ${\omega }_{h}$ and the coefficient *α*

	Baseline		Follow-up	
	${\omega }_{h}$	Coefficient *α*	${\omega }_{h}$	Coefficient *α*
Anxiety	0.87	0.94	0.92	0.96
Depression	0.87	0.94	0.89	0.96
Fatigue	0.84	0.90	0.86	0.91
Pain Interference	0.94	0.98	0.96	0.98
Pain Intensity	0.91	0.90	0.92	0.92
Sleep Disturbance	0.82	0.92	0.82	0.93

### Identifying patients who had reliable change

Table 3 shows the number and percentages of patients classified as the same, worse, or better by CTT and IRT approaches. Across six domains, we found that CTT and IRT approaches to estimating reliable change agree on the classifications of changes the majority of the time (80% to 98%). When there were disagreements, RCI_IRT_ tended to identify more patients as having changed in their symptoms while RCI_CTT_ suggested that the patients had no reliable change. Sometimes, RCI_CTT_ detected changes that RCI_IRT_ categorized as stable. How can we explain these disagreements?

**Table 3:** Cross tabulations of detection of statistical reliable change by CTT and IRT

			RCI_IRT_	
	**Anxiety (n = 1,105)**	Worse	Same	Better
RCI_CTT_	Worse	215 (19%)	11 (1%)	0
	Same	48 (4%)	643 (58%)	40 (4%)
	Better	0	17 (2%)	131 (12%)
	**Depression (n = 1,162)**	Worse	Same	Better
	Worse	190 (16%)	40 (3%)	0
	Same	73(6%)	677 (58%)	33 (3%)
	Better	0	46 (4%)	103 (9%)
	**Fatigue (n = 1,140)**	Worse	Same	Better
	Worse	262 (23%)	0	0
	Same	118 (10%)	582 (51%)	79 (7%)
	Better	0	1 (0.1%)	98 (9%)
	**Pain Interference (n = 1,090)**	Worse	Same	Better
	Worse	245 (22%)	26 (2%)	0
	Same	54 (5%)	515 (47%)	29 (3%)
	Better	0	39 (4%)	182 (17%)
	**Pain Intensity (n = 1,144)**	Worse	Same	Better
	Worse	235 (20.5%)	17 (1.5%)	0
	Same	0	693 (60.6%)	0
	Better	0	9 (0.8%)	190 (16.6%)
	**Sleep Disturbance (n = 1,089)**	Worse	Same	Better
	Worse	185 (16.9%)	0	0
	Same	93 (9%)	624 (57%)	86 (8%)
	Better	0	1 (0.1%)	100 (9%)

The parenthesis next to each domain name contains the denominator for calculating the percentages

**Fig. 1:**
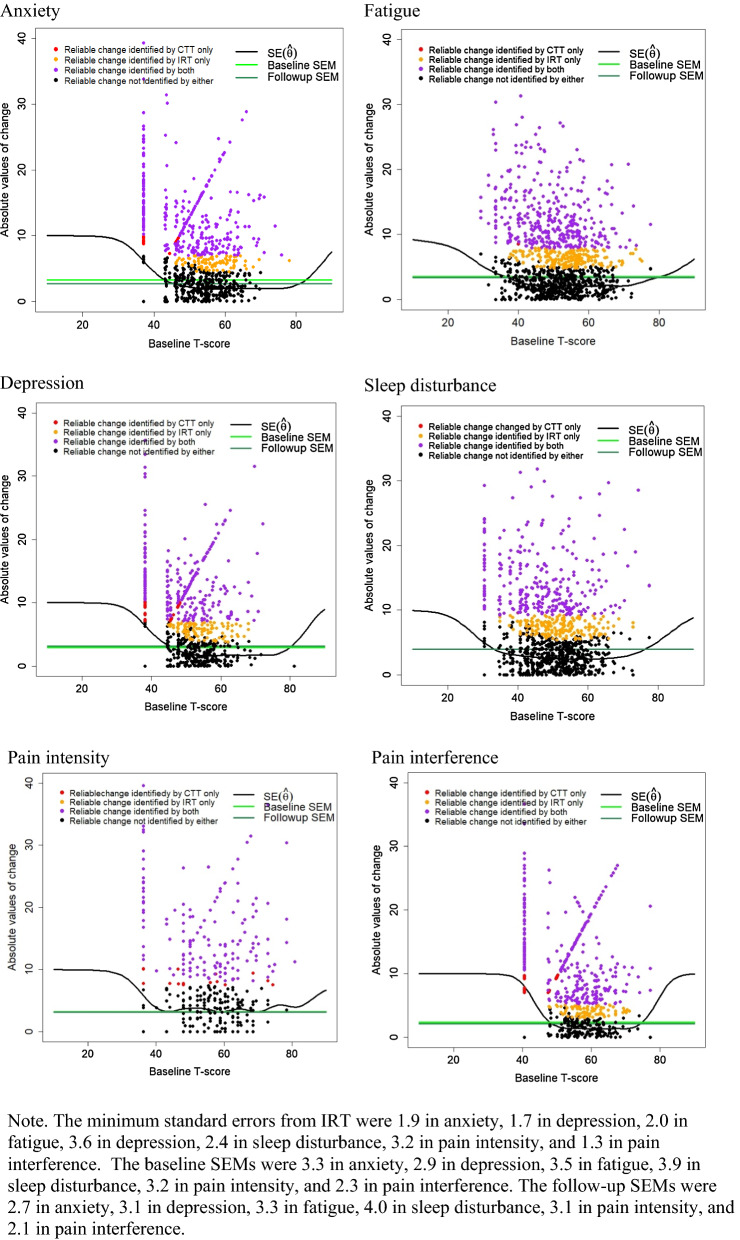
Absolute change conditional on baseline scores (T score metric) plotted against the standard errors of estimation (IRT) and the pooled standard errors of measurement (CTT). The dots represent absolute values of change scores

**Fig. 2:**
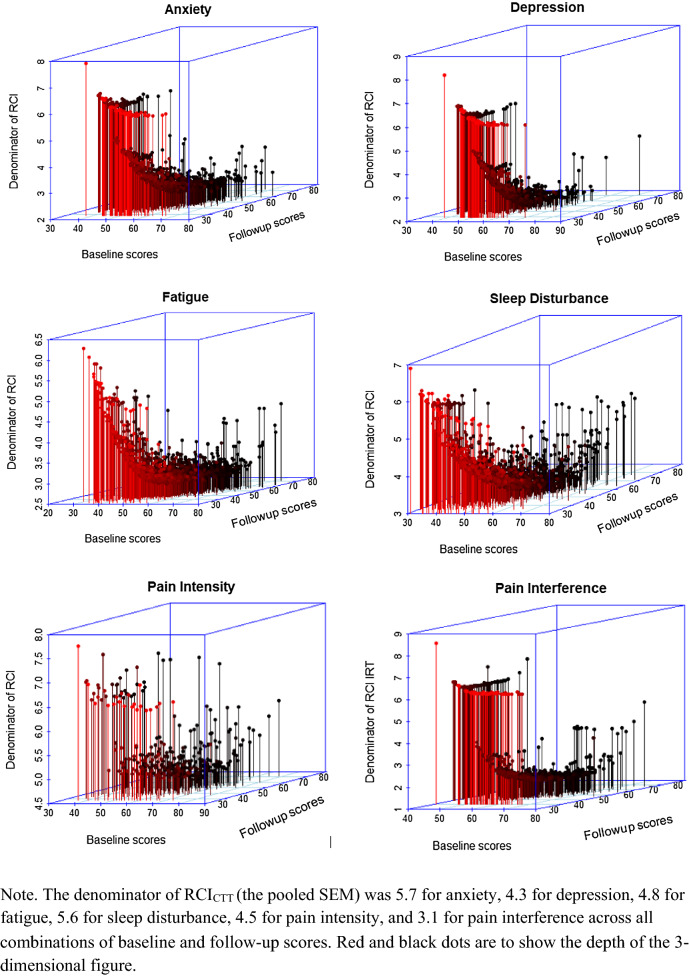
The denominator of RCI_IRT_ (in formula 2) as a function of the baseline and follow-up scores

Figure 1 shows how identification rates are related to the standard errors. Typically for *T* scores greater than 45, the measurement error in the IRT approach is consistently lower than for the CTT approach. In most cases, if a person was categorized as worsened (or improved) by the CTT approach, they were necessarily classified as worsened (or improved) by the IRT approach. There were some instances in which a patient was classified as stable according to the IRT approach but worsening/improving by the CTT approach. When this happened, scores at baseline or follow-up were in the range where 1.65 × SE($\widehat{\theta }$) exceeded 1.65 × SEM. For example, for depression, there were 86 individuals (red dots in Fig. 1) who were classified as stable by IRT but changing by CTT. These patients had either their baseline or follow-up scores lower than 40, and their scores at both time points were lower than 50. Figure 2 shows the relationship between the scores and the denominator of RCI_IRT_.

### Magnitude of the reliable change scores based on CTT and IRT

Table 4 presents the amount of change in *T* scores corresponding to an RCI value of 1.65 using the two methods. The amount of change in *T* scores according to RCI_CTT_ of 1.65 was 7.03 in anxiety, 7.08 in depression, 7.95 in fatigue, 9.22 in sleep disturbance, 7.40 in pain intensity, and 5.14 in pain interference, which were constant across the score range by definition of CTT. The amount of change in *T* scores for RCI_IRT_ of 1.65 varied across the score range, by definition of IRT, with many of the RCI_IRT_ estimates being below those for RCI_CTT_, particularly around and above the center of the *T* score distribution. For pain intensity, on the other hand, IRT standard errors tended to be greater than SEM across the score range, so patients had to score higher to be classified as having changed according to RCI_IRT_. This is shown by the red dots (reliable change identified by CTT only) scattered across score range for pain intensity in Fig. 1.

**Table 4:** T score changes corresponding to RCI value of 1.65

	Baseline Score	CTT	IRT
Anxiety	35	7.0	12.7
	40		8.4
	45		5.8
	50		4.9
	55		4.5
	60		4.5
	65		4.5
	70		4.5
	75		5.2
Depression	35	7.1	13.9
	40		9.4
	45		6.1
	50		4.4
	55		3.8
	60		4.0
	65		4.0
	70		4.0
	75		7.4
Fatigue	30	8.0	9.5
	35		7.3
	40		5.7
	45		5.0
	50		4.9
	55		4.9
	60		4.9
	65		4.9
	70		5.7
	75		7.8
Sleep Disturbance	30	9.2	9.8
	35		7.5
	40		6.5
	45		6.0
	50		5.7
	55		5.7
	60		5.7
	65		6.4
	70		9.2
	75		9.9
Pain Intensity	40	7.4	8.8
	45		8.2
	50		9.2
	55		8.4
	60		7.6
	65		8.7
	70		8.6
Pain Interference	40	5.1	14.0
	45		7.1
	50		3.6
	55		3.2
	60		3.0
	65		3.0
	70		8.7
	75		10.0

### Identifying patients who had reliable change and meaningful change based on change in PRO-CTCAE categorical responses

Table 5 presents detailed information on the number of patients who had statistically reliable improvement or deterioration based on RCI_CTT_ and RCI_IRT_ and meaningful improvement or deterioration based on the change in PRO-CTCAE categorical responses. Table 5 decomposes the number of patients who had reliable and meaningful change according to PRO-CTCAE. Each row indicates, the number of people who changed in absolute values, people who reliably or meaningfully changed, and the summary statistics of their scores. The table also reports these numbers separately for CTT versus IRT, and for worsening versus improvement. There were very few missing PRO-CTCAE scores in general (0% to 5% depending on domains). The percentages of patients who had reliable change, and subsequently meaningful change are reported for each domain below. The minimum observed score that was classified as meaningful (i.e., minimally important difference) according to the criterion used in this study reflected the T scores corresponding to RCI value of 1.65 shown in Table 4.

**Table 5:** Frequency of patients who had reliable and meaningful worsening and improvement, and the summary statistics of their change scores

	Number of patients who became worse in absolute values (N)	CTT			IRT		
		**Reliably worse (N)**	**Meaning-fully worse (N)**^**1**^	Summary statistics of the change scores of those who became **meaningfully worse** (min., median, max.)	**Reliably worse (N)**	**Meaning-** **fully worse (N)**	Summary statistics of the change scores of those who became **meaningfully worse** (min., median, max.)
Anxiety	502	226	152	7.1, 13.4, 39.4	263	165	4.5, 12.9, 39.4
Depression	493	230	153	7.1, 12.6, 35.7	263	169	4.0, 11.6, 35.7
Fatigue	712	262	195	8.0, 13.0, 31.3	380	251	4.8, 11.6, 31.3
Sleep Disturbance	590	185	150	9.3, 13.7, 31.9	278	208	5.4, 11.7, 31.9
Pain Intensity	433	252	198	7.5, 13.7, 45.6	235	187	8.2, 13.7, 45.6
Pain Interference	439	271	226	5.4, 13.5, 36.7	299	242	3.1, 12.9, 36.7

	Number of patients who became better in absolute values (N)	CTT			IRT		
		**Reliably better (N)**	**Meaning-fully better (N)**	Summary statistics of the change scores of those who became **meaningfully better** (min., median, max.)	**Reliably better (N)**	**Meaning-** **fully better (N)**	Summary statistics of the change scores of those who became **meaningfully better** (min., median, max.)
Anxiety	401	148	104	−28.9, −11.9, −7.1	171	122	−28.9, −10.7, −4.8
Depression	372	149	93	−31.5, −11.4, −7.1	136	88	−31.5, −11.8, −3.8
Fatigue	415	99	66	−23.1, −10.6, −8.0	177	112	−23.1, −8.4, −4.8
Sleep Disturbance	450	101	70	−29.8, −12.6, −9.3	186	116	−29.8, −10.0, −5.5
Pain Intensity	334	199	151	−36.5, −12.6, −7.7	190	144	−36.5, −13.1, −8.2
Pain Interference	335	221	157	−27.1, −13.0, −5.3	211	154	−27.1, −13.0, −3.0

*Anxiety* There were 502 patients who deteriorated in *T* scores (*T* score change > 0). Among these patients, 226 (45%) had reliable worsening based on RCI_CTT_; among 226, 152 (67%) had meaningful worsening based on change in PRO-CTCAE. Based on RCI_IRT_, 263(52%) had reliable worsening; 165 (63%) had meaningful worsening.

There were 401 patients who improved in *T* scores (*T* score change < 0). Among 401, 148 (37%) had reliable improvement based on CTT; among 148, 104 (70%) also had meaningful improvement. Based on IRT, 171 (43%) had reliable improvement; Among those with reliable improvement, 122 (71%) also had meaningful improvement.

*Depression* There were 493 patients who deteriorated in *T* scores: 230 (47%) had reliable worsening based on CTT; among 230 patients, 153 (67%) also had meaningful worsening. Based on IRT, 263 (53%) had reliable worsening; among 263 patients, 169 (64%) also had meaningful change.

There were 372 patients who improved in *T* scores (*T* score change < 0). Among 372, 149 (40%) had reliable improvement based on CTT; among 149, 93 (62%) had meaningful improvement. Based on IRT, 136 (37%) had reliable improvement; Among those with reliable improvement, 88 (65%) had meaningful improvement.

*Fatigue* There were 712 patients who deteriorated in *T* scores: 262 (37%) had reliable worsening based on CTT; among 262 patients, 195 (74%) also had meaningful worsening. Based on IRT, 380 (53%) had reliable worsening; among 380 patients, 251 (66%) also had meaningful change.

There were 415 patients who improved in *T* scores (*T* score change < 0). Among 415, 99 (24%) had reliable improvement based on CTT; among 99, 66 (67%) had meaningful improvement based on RCI_CTT_. Based on IRT, 177 (43%) had reliable improvement; Among those with reliable improvement, 112 (63%) had meaningful improvement.

*Sleep disturbance* There were 590 patients who deteriorated in *T* scores: 185 (31%) had reliable worsening based on CTT; among 185 patients, 150 (81%) also had meaningful worsening. Based on IRT, 278 (47%) had reliable worsening; among 278 patients, 208 (75%) also had meaningful change.

There were 450 patients who improved in *T* scores (*T* score change < 0). Among 450, 101 (22.4%) had reliable improvement based on CTT; among 101, 70 (69%) had meaningful improvement. Based on IRT, 186 (41%) had reliable improvement; Among those with reliable improvement, 116 (62%) had meaningful improvement.

*Pain Intensity* Among 433 patients who deteriorated in *T* scores, 252 (58%) had reliable worsening; among 252, 198 (79%) had meaningful worsening using the CTT method. Using the IRT method, 235 (54%) had reliable worsening; among 235, 187 (80%) meaningful worsening.

Among 334 patients who improved in *T* scores, 199 (60%) had reliable improvement among 199, 151 (76%) had meaningful improvement using the CTT method. Using the IRT method, 190 (57%) had reliable improvement; among 190, 144 (76%) meaningful improvement.

*Pain interference* Among 439 patients who deteriorated in *T* scores, 271 (62%) had reliable worsening; among 271, 226 (83%) had meaningful worsening using the CTT method. Using the IRT method, 299 (68%) had reliable worsening; among 299, 242 (81%) meaningful worsening.

Among 335 patients who improved in *T* scores, 221 (66%) had reliable improvement and 157 (71%) had meaningful improvement using the CTT method. Using the IRT method, 211 (63%) had reliable improvement; among 211, 154 (73%) meaningful improvement.

## Discussion

Prior studies focusing on classification rates between RCI_CTT_ and RCI_IRT_ showed consistent classification between RCI_CTT_ and RCI_IRT_ in most patients (e.g., About 78% to 92% in [7; 8; 10]). The current study showed, in PROMIS short forms on core symptoms in cancer patients, RCI_CTT_ and RCI_IRT_ agree on the classifications of changes 83% to 98% of the times. We also demonstrated how differences in standard errors in relation to the score distributions result in differing classification decisions for an individual by IRT and CTT in PROMIS measures. When there were disagreements such that CTT could not detect changes that were detected by IRT, they occurred when measurement errors were overestimated by CTT, where scores at both timepoints were in the range of 43 to 78 in anxiety, 45 to 70 in depression, 38 to 80 in fatigue, 35 to 78 in sleep disturbance, and 48 to 74 in pain interference. Accurate measurement would be important in these ranges, because they include non-trivial symptom levels that physicians or patients may not want to ignore. The CTT method would sometimes detect changes in the extreme score range not detected by IRT, which shows CTT method may be misclassifying stable scores as changing by underestimating error in that score range. For example, RCI_CTT_ detected changes which RCI_IRT_ classified as stable when scores at both timepoints were ≤ 47 in anxiety, ≤ 48 in depression, ≤ 40 in fatigue, ≤ 40 in sleep disturbance, and ≤ 50 in pain interference (Appendix). The appendix shows that CTT may classify patients as changing although the information of the scale does not allow for such classification in the specific score range. Should we worry about PROMIS short forms not reaching desired level of precision in those score ranges? For screening purpose, physicians are unlikely to be worried about pretest and posttest PROMIS symptom scores ≤ 50, because this indicates the patient is scoring better than or equal to the population norm.

Hays et al. [8] used the 4-item scales in PROMIS-29 and found CTT classified 21% as changing in emotional distress but IRT indicating no change. One may wonder the reasons for larger proportions of patients being classified as changing only by CTT in their study compared to 3 to 7% in anxiety and depression in the current study. The four items used in the emotional distress scale in Hays et al. [8] were also in the Anxiety 8a in the current study. The other four items used in the emotional distress scale were also in the Depression 8a used in the current study. Both studies used EAP for standard error estimation. Their reliability estimates were 0.86 and 0.9, close in values to ${\omega }_{h}$’s in the current study. Based on these similarities, the major difference from the current study may be attributed to (1) the test length: The prior study used standard error estimates based on four items (average of standard errors for the 4-item depression and those for the 4-item anxiety scales), which would raise the SE($\widehat{\theta }$) shown in Anxiety or Depression in Fig. 1 higher, and (2) the sample score distributions: If there were many patients whose emotional distress level was at the lower side at both time points (e.g., below the population norm) where information is lower, then RCI_CTT_ may have been overly optimistic about detecting changes in these patients.

This study collected responses from a large and diverse sample of patients recruited from multiple cancer centers with a variety of cancer types and stages, as well as investigating a variety of core symptom domains with PROMIS short forms. Of note, this study used patient perspectives for identifying meaningful change in PROMIS short forms. Statistically reliable change alone may not communicate whether patients also find the change meaningful. On the other hand, using a criterion only for meaningful change but not reliable change can result in logical contradiction. For example, in fatigue short form, patients whose PRO-CTCAE scores improved had PROMIS change scores ranging from -23 to 13: This range includes 0 (no change) to 13 (worsening).

A limitation of the current study is that we have not investigated the reliable change in CAT. Although the thresholds derived for reliable change from this study would be largely generalizable to cancer population using PROMIS in their respective domains, further research can be conducted using RCI with SE($\widehat{\theta }$) for PROMIS administered with CAT to see whether changes can be better identified at the lower symptom levels. As the electronic health records (EHR) facilitate longitudinal collection of PRO data, a data field containing whether the RCI_IRT_ exceeds a critical value may provide useful information on reliable worsening or improvement in addition to the *T* scores themselves.

For questionnaires developed with CTT methods, the RCI_CTT_ can also be implemented in EHR. We showed (Fig. 1) that baseline and follow-up SEMs were either extremely close or equal, which suggests that computing RCI based on the SEM of the pretest only would not have biased the results. This has an implication for large-scale questionnaires created with CTT methods and implemented in EHR. Because we do not need separate follow-up SEM, a data field containing whether the RCI_CTT_ exceeds a critical value can be populated, immediately after the patient completes a follow-up questionnaire. One limitation for RCI_CTT_ would be that unlike an IRT-based measure that had been calibrated on the U.S. general population, SEM is a more sample-dependent statistic. Just as accurate estimates of all parameters are required from the IRT methods to detect changes, accurate estimates of SEM would be necessary to determine whether an observed change in a new patient is a reliable change.

The current study used *T-*scores to compute change scores in both CTT and IRT methods, because *T*-scores are preferred over raw summed scores for the PROMIS measures and we used the metric that would be common when reporting PROMIS scores. It should be noted that *T*-scores are IRT-based scores. Because studies [25–27] have reported reliable change in PROMIS using RCI with CTT-based standard errors despite using IRT-based scores, we used the same approach when we applied the CTT method.

We demonstrated how categorical evaluation of patients’ self-reported adverse events can be used for detecting meaningful change in cancer patients’ symptoms in PROMIS. A limitation for the current approach is that one would need to administer an additional item asking categorical evaluation of their symptoms at each time point to determine whether the reliable change was also meaningful. Furthermore, the assumption that a 1-level change in PRO-CTCAE is meaningful can be tested in a qualitative study. Cut-scores for PROMIS item bank in anxiety, depression, fatigue, and pain interference have been developed from clinician judgment with bookmarking method [28]. A future study can investigate the detection of reliable and meaningful change in relation to cut-scores, as well as interpreting the change in PRO scores in concert with other aspects of an individual’s situation (e.g., trajectory of illness and treatment, personal and social life circumstances, or goals and values). Applying the two-step criteria demonstrated in this study allows determining which individual cases changed reliably, provides a straightforward evaluation of meaningfulness of the change, and facilitates interpretability and communication of PRO results.

## Conclusions

The current study demonstrated how two approaches, CTT and IRT, for calculating RCI converge or diverge in assessing individual-level change in PROMIS short forms on core symptoms experienced by cancer patients. The interpretation of change scores should take into account the standard errors that differ across the range of the scores whenever possible. We derived the thresholds for reliable change at different levels of baseline scores for investigators using the PROMIS instruments in oncology. We derived percentages of patients who had reliable and meaningful change as reference values for designing clinical trials.

## Supplementary Information

Below is the link to the electronic supplementary material.

Supplementary file1 (PDF 367 KB)

## Data Availability

Data can be made available upon reasonable request to the senior author. All requests will be reviewed.
