# Supplementary material for: Identifying meaningful change on PROMIS short forms in cancer patients: a comparison of item response theory and classic test theory frameworks
Source: Qual Life Res. 2022 Sep 24;32(5):1355–67. doi: 10.1007/s11136-022-03255-3 (PMC10123030; doi:10.1007/s11136-022-03255-3)
Supplement: Supplementary file 1 — Supplementary file1 (PDF 367 KB) [file 11136_2022_3255_MOESM1_ESM.pdf]

Appendix. Example of actual patient responses where IRT classified patients as not reliably changing but CTT as reliably changing.

A. Anxiety

Baseline score of 37	Follow-up score of 47
1. I felt fearful. **Never**	1. I felt fearful. **Never**
2. I found it hard to focus on anything other than my anxiety. **Never**	2. I found it hard to focus on anything other than my anxiety. **Rarely**
3. My worries overwheled me. **Never**	3. My worries overwheled me. **Rarely**
4. I felt uneasy. **Never**	4. I felt uneasy. **Never**
5. I felt nervous. **Never**	5. I felt nervous. **Never**
6. I felt like I needed help for my anxiety. **Never**	6. I felt like I needed help for my anxiety. **Never**
7. I felt anxiety. **Never**	7. I felt anxiety. **Never**
8. I felt tense. **Never**	8. I felt tense. **Never**

B. Depression

Baseline score of 38	Follow-up score of 48
1. I felt worthless. **Never**	1. I felt worthless. **Never**
2. I felt helpless. **Never**	2. I felt helpless. **Never**
3. I felt depressed. **Never**	3. I felt depressed. **Never**
4. I felt hopeless. **Never**	4. I felt hopeless. **Rarely**
5. I felt like a failure. **Never**	5. I felt like a failure. **Never**
6. I felt unhappy. **Never**	6. I felt unhappy. **Never**
7. I felt that I had nothing to look forward to. **Never**	7. I felt that I had nothing to look forward to. **Rarely**
8. I felt that nothing could cheer me up. **Never**	8. I felt that nothing could cheer me up. **Never**

C. Fatigue

Baseline score of 29	Follow-up score of 40
1. How often did you feel tired? **Never**<br>2. How often did you experience Extreme exhaustion? **Never**<br>3. How often did you run out of energy? **Never**<br>4. How often did your fatigue limit You at work (include work at home)? **Never**<br>5. How often were you too tired to think clearly? **Never**<br>6. How often were you too tired to take a bath or shower? **Never**<br>7. How often did you have enough energy to get things done due to fatigue? **Always**<br>8. How often did you have to push yourself to get things done due to fatigue? **Never**<br>9. How often did you have trouble Finishing things due to fatigue? **Never**	1. How often did you feel tired? **Sometimes**<br>2. How often did you experience Extreme exhaustion? **Never**<br>3. How often did you run out of energy? **Never**<br>4. How often did your fatigue limit You at work (include work at home)? **Never**<br>5. How often were you too tired to think clearly? **Never**<br>6. How often were you too tired to take a bath or shower? **Never**<br>7. How often did you have enough energy to get things done due to fatigue? **Never**<br>8. How often did you have to push yourself to get things done due to fatigue? **Never**<br>9. How often did you have trouble Finishing things due to fatigue? **Never**

Note. The item, “How often did you have enough energy to get things done due to fatigue” had low correlations (Spearman’s correlation: 0.05 to 0.16) with the other items due to the scoring scale that runs in the opposite direction.

D. Sleep disturbance

Baseline score of 31	Follow-up score of 40
1. My sleep quality was **Very good**<br>2. My sleep was refreshing. **Very much**<br>3. I had a problem with my Sleep. **Not at all**<br>4. I had difficulty falling asleep. **Not at all**<br>5. My sleep was restless. **Not at all**<br>6. I tried hard to get to sleep **Not at all**<br>7. I worried about not being able to fall asleep **Not at all**<br>8. I was satisfied with my sleep **Very much**	1. My sleep quality was **Good**<br>2. My sleep was refreshing. **Very much**<br>3. I had a problem with my Sleep. **A little bit**<br>4. I had difficulty falling asleep. **Not at all**<br>5. My sleep was restless. **A little bit**<br>6. I tried hard to get to sleep **Not at all**<br>7. I worried about not being able to fall asleep **Not at all**<br>8. I was satisfied with my sleep **Quite a bit**

E. Pain interference

Baseline score of 41	Follow-up score of 50
1. How much did pain interfere with Your day to day activities? **Not at all**<br>2. How much did pain interfere with Work around the home? **Not at all**<br>3. How much did pain interfere with your ability to participate in social activities? **Not at all**<br>4. How much did pain interfere with your household chores? **Not at all**<br>5. How much did pain interfere with the things you usually do for fun? **Not at all**<br>6. How much did pain interfere with your enjoyment of social activities? **Not at all**<br>7. How much did pain interfere with your enjoyment of life? **Not at all**<br>8. How much did pain interfere with your family life? **Not at all**	1. How much did pain interfere with Your day to day activities? **A little bit**<br>2. How much did pain interfere with Work around the home? **A little bit**<br>3. How much did pain interfere with your ability to participate in social activities? **Not at all**<br>4. How much did pain interfere with your household chores? **Not at all**<br>5. How much did pain interfere with the things you usually do for fun? **Not at all**<br>6. How much did pain interfere with your enjoyment of social activities? **Not at all**<br>7. How much did pain interfere with your enjoyment of life? **Not at all**<br>8. How much did pain interfere with your family life? **Not at all**

F. Pain interference

Baseline score of 40.5	Follow-up score of 50.3
1. How much did pain interfere with Your day to day activities? **Not at all**<br>2. How much did pain interfere with Work around the home? **Not at all**<br>3. How much did pain interfere with your ability to participate in social activities? **Not at all**<br>4. How much did pain interfere with your household chores? **Not at all**<br>5. How much did pain interfere with the things you usually do for fun? **Not at all**<br>6. How much did pain interfere with your enjoyment of social activities? **Not at all**<br>7. How much did pain interfere with your enjoyment of life? **Not at all**<br>8. How much did pain interfere with your family life? **Not at all**	1. How much did pain interfere with Your day to day activities? **A little bit**<br>2. How much did pain interfere with Work around the home? **A little bit**<br>3. How much did pain interfere with your ability to participate in social activities? **Not at all**<br>4. How much did pain interfere with your household chores? **Not at all**<br>5. How much did pain interfere with the things you usually do for fun? **Not at all**<br>6. How much did pain interfere with your enjoyment of social activities? **Not at all**<br>7. How much did pain interfere with your enjoyment of life? **Not at all**<br>8. How much did pain interfere with your family life? **Not at all**
